# Expanding the clinical and molecular spectrum of Schaaf-Yang syndrome in China: evidence for a severe phenotypic subgroup associated with *MAGEL2* c.1996-Region variants

**DOI:** 10.3389/fcell.2026.1932244

**Published:** 2026-09-30

**Authors:** Xue-Fei Chen, Shu-jiong Mao, Sheng Zhong, Shanshan Shen, Xue-Yong Lou, Chao-Chun Zou, Tao Ling

**Affiliations:** 1 Department of Endocrinology and Metabolism, Jinhua Municipal Central Hospital, Jinhua, China; 2 Children’s Hospital, Zhejiang University School of Medicine, Hangzhou, China; 3 Department of Pediatrics, Affiliated Hangzhou First People’s Hospital, School of Medicine, Westlake University, Hangzhou, China; 4 Department of Gynecology, Jinhua Maternity and Child Health Care Hospital, Jinhua, China; 5 Department of Endocrinology, The Children’s Hospital of Zhejiang University School of Medicine, National Clinical Research Center for Children and Adolescents’ Health and Diseases, Hangzhou, China; 6 Department of Gastroenterology, Jinhua Municipal Central Hospital, Jinhua, China

**Keywords:** Schaaf-Yang syndrome, MAGEL2, imprinting inheritance, truncating variants, respiratory dysfunction

## Abstract

**Introduction:**

Schaaf-Yang syndrome (SYS) is a rare imprinting-related neurodevelopmental disorder caused by pathogenic variants in the *MAGEL2* gene. Although the clinical spectrum of SYS has been increasingly recognized, data from Chinese patients remain limited.

**Methods:**

We conducted a retrospective questionnaire-based case series to delineate the clinical and molecular characteristics of Chinese individuals with MAGEL2-related disorders among evaluable individuals.

**Results:**

We present 28 individuals harboring *MAGEL2* variants. Developmental delay or intellectual disability was universal. The cohort exhibited the expected multisystem SYS phenotypes, including neonatal hypotonia, feeding difficulties, respiratory involvement, congenital joint contractures, dysmorphic features, endocrine abnormalities, and neurobehavioral manifestations. We identified 23 distinct *MAGEL2* variants, including 15 novel variants. Fifteen patients carried *de novo* variants, while 13 had paternally inherited variants, consistent with the imprinting mechanism of SYS. In addition to truncating variants, eight individuals carried missense variants, and their phenotypes appeared to be at the mild end of the SYS spectrum. The *MAGEL2* c.1996 site was a mutational hotspot, with variants affecting this locus identified in seven patients, including five with the recurrent c.1996dupC variant. Four patients harboring variants at the c.1996 site died of respiratory failure during infancy.

**Discussion:**

This study provides the first systematic characterization of Chinese patients with SYS and expands the clinical and molecular spectrum of MAGEL2-related disease. Our findings identify early respiratory dysfunction as a major determinant of disease severity and suggest that truncating variants affecting the *MAGEL2* c.1996 site might be associated with more severe phenotypes and potentially an increased risk of early mortality. This exploratory observation requires confirmation in larger longitudinal cohorts. Recognition of this genotype–phenotype pattern may help guide early molecular diagnosis, risk assessment, and clinical management in the early life stage of individuals with SYS.

## 1 Introduction

Schaaf-Yang syndrome (SYS; OMIM #615547) is a rare imprinting-related neurodevelopmental disorder caused by pathogenic variants in the *MAGEL2* gene, which lies within the Prader-Willi syndrome (PWS, OMIM #176270) locus on chromosome 15 (Schaaf et al., 2013; Mejlachowicz et al., 2015; Juriaans et al., 2022). Its clinical spectrum commonly includes neonatal hypotonia, developmental delay/intellectual disability (DD/ID), autism spectrum disorder (ASD) and congenital joint contractures (Fountain et al., 2017; Mccarthy et al., 2018; Heimdörfer et al., 2024). The disorder was first delineated in 2013, when truncating variants on the paternal allele of *MAGEL2* were identified in four individuals with Prader-Willi-like features (Schaaf et al., 2013). The diagnosis of SYS is confirmed by molecular genetic testing through identification of a heterozygous pathogenic variant affecting the paternally derived *MAGEL2* allele (Schaaf et al., 1993).

MAGEL2 (melanoma antigen L2) belongs to a MAGE protein family of E3 RING ubiquitin ligase regulators (Tacer et al., 2017; Hao et al., 2013; Doyle et al., 2010). In the MUST complex, MAGEL2 brings together the RING-type E3 ubiquitin transferase TRIM27 and the ubiquitin-specific protease USP7 at retromer-positive endosomes, allowing local control of WASH-associated actin assembly and cargo recyclings (Hao et al., 2013; Doyle et al., 2010; Lee and Potts, 2017). MAGEL2 is highly expressed in the central nervous system, predominantly present in the hypothalamus (Lee et al., 2000). MAGEL2 is thought to act as a multifunctional hypothalamic and neurodevelopmental regulatory protein, linking retromer trafficking, neuropeptide biology, and neurite development with the control of endocrine homeostasis, feeding behavior, circadian regulation, and adaptive responses to environmental signals (Hoyos Sanchez et al., 2023; Schubert and Schaaf, 2024). Recent researches have proposed a new perspective for the pathogenic mechanism of SYS. Rather than acting solely through haploinsufficiency, SYS-associated truncating variants may produce truncated proteins with altered subcellular behavior and neomorphic effects (Fountain et al., 2017; Hug et al., 2016; Lejeune, 2022). This mechanism provides a plausible explanation for the distinct SYS phenotypes and for genotype-dependent differences in severity, although further functional studies are required to define how individual variant perturb MAGEL2-associated networks (Maaß et al., 2023; Camerino, 2024).

As a maternally imprinted and paternally expressed gene, the clinical consequence of *MAGEL2* gene depends on its parental origin: variants transmitted through the maternal allele may pass silently across generations, whereas the variant on the paternal allele is expected to be disease-causing (Fountain et al., 2017; Guo et al., 2019). The penetrance of SYS is considered to be 100% (Schaaf et al., 1993). Approximately half of the reported *MAGEL2* variants are paternally inherited from an unaffected carrier father, whereas the remainder occur *de novo*. For accurate genetic counseling and recurrence-risk estimation, targeted testing of the father for the *MAGEL2* variant found in his offsprings is recommended. When the variant is absent from paternal blood, methylation-sensitive or other allele-origin assays should be considered to confirm whether the proband’s variant is located on the paternal allele (Schubert and Schaaf, 2024). Although most genetically confirmed SYS cases are caused by truncating or nonsense variants in *MAGEL2* (Schaaf et al., 2013), two missense variants have been reported in individuals with MAGEL2-related phenotypes, including p. Ala538Glu (Patak et al., 2019) and the recently described p. Pro422Leu (Pastucha et al., 2026). The phenotypic spectrum of the two cases appears milder, with fewer clinical manifestations than those typically observed in patients with truncating mutations.

Patients with SYS and PWS share overlapping clinical phenotypes, including neonatal hypotonia, feeding difficulties, intellectual disability, developmental delay, and hypogonadism (Fountain and Schaaf, 2016). Nevertheless, SYS is more strongly associated with joint contractures and shows a substantially greater burden of ASD (Bennett et al., 2015; Gregory et al., 2019). By contrast, hyperphagia and morbid obesity appear to be less common in SYS than in PWS (Cassidy et al., 2012). Notably, patients with SYS tend to show more severe growth and cognitive impairment than individuals with PWS (Mccarthy et al., 2018; Saitoh, 2022), whereas impulsive and compulsive behaviors are reported less frequently (Thomason et al., 2018). Compared with PWS, which has a relatively higher prevalence, SYS has been rarely reported, and its prognosis remains largely unexplored. Nevertheless, fatal cases of SYS have been documented. Mejlachowicz et al. (Mejlachowicz et al., 2015) reported 3 children with SYS who presented with lethal arthrogryposis multiplex congenita (AMC) and reduced fetal mobility, all died at the fetal stage. Fountain et al. (Fountain et al., 2017) described 2 fetuses with lethal arthrogryposis and akinesia, and the corresponding pregnancies were electively terminated at 21 and 14 weeks of gestation, respectively. In our previous study, we reported two patients with SYS who died of respiratory failure during infancy (Chen et al., 2020).

Given the rarity of SYS and the relatively recent establishment of its disease concept, the condition is likely underrecognized in China, and the true number of affected individuals remains unclear. To better characterize SYS in the Chinese population, we conducted a nationwide questionnaire-based survey to collect data of cases with *MAGEL2* gene variants, including cases with fatal outcomes. This approach aimed to delineate the diagnostic status, clinical spectrum, and genetic characteristics of SYS in Chinese patients.

## 2 Materials and methods

We conducted a retrospective questionnaire-based case series of Chinese individuals with genetically confirmed or clinically suspected SYS. Informed consent was obtained from the parents or legal guardians of all participants for publication of clinical information, pedigrees and genetic findings. All of the individuals have undergone trio-based whole exome sequencing (trio-WES). Candidate *MAGEL2* variants were confirmed by Sanger sequencing in the probands and available relatives. Clinical and molecular data were collected using a structured survey and review of available medical records, including sex, age at evaluation, perinatal history, neurodevelopmental features, feeding and respiratory manifestations, musculoskeletal abnormalities, endocrine findings, neurobehavioral features, survival status, and so on. Each phenotype was coded as present, absent, not assessed or not applicable. Frequency denominators included only participants with an age-appropriate and sufficiently documented assessment. Short stature was defined as length or height below −2 SD for age and sex, and scoliosis as a documented clinical or radiographic finding. Sleep apnea required polysomnographic confirmation or a documented specialist diagnosis and was distinguished from nonspecific neonatal apnea. Growth hormone deficiency and hypogonadism were counted only when evaluated using age-appropriate endocrine criteria.

Variants were described according to HGVS nomenclature using transcript NM_019066.5. All *MAGEL2* variants were interpreted according to the 2015 ACMG guidelines using population-frequency, predicted molecular consequence, segregation, parent-of-origin, phenotype-concordance, and available database and literature evidence. Variants were classified as pathogenic (P), likely pathogenic (LP), of uncertain significance (VUS), likely benign, or benign. Variant types, recurrent sites, and genotype–phenotype associations were summarized descriptively, with emphasis on truncating variants, missense variants, mosaic variants, and variants involving the c.1996 region. The study was conducted in accordance with the Declaration of Helsinki, and approved by the Ethics Committee of Jinhua Central Hospital (approval no. 20263340101).

## 3 Results

### 3.1 Cohort overview

We identified 28 patients with *MAGEL2* variants from 28 families (Figure 1). The cohort comprised 19 males and 9 females, with ages ranging from the neonatal period to childhood (3 days-6 years; median, 17 months). Four patients died due to respiratory distress. Clinical manifestations of this cohort often emerge early in life, with a complex phenotype that may include feeding difficulties, respiratory compromise, hypotonia, distal contractures, neuromuscular symptoms, endocrine abnormalities, and dysmorphic features. Additional clinical issues may develop later and involve multiple organ systems, underscoring the need for coordinated multidisciplinary management.

**FIGURE 1:**
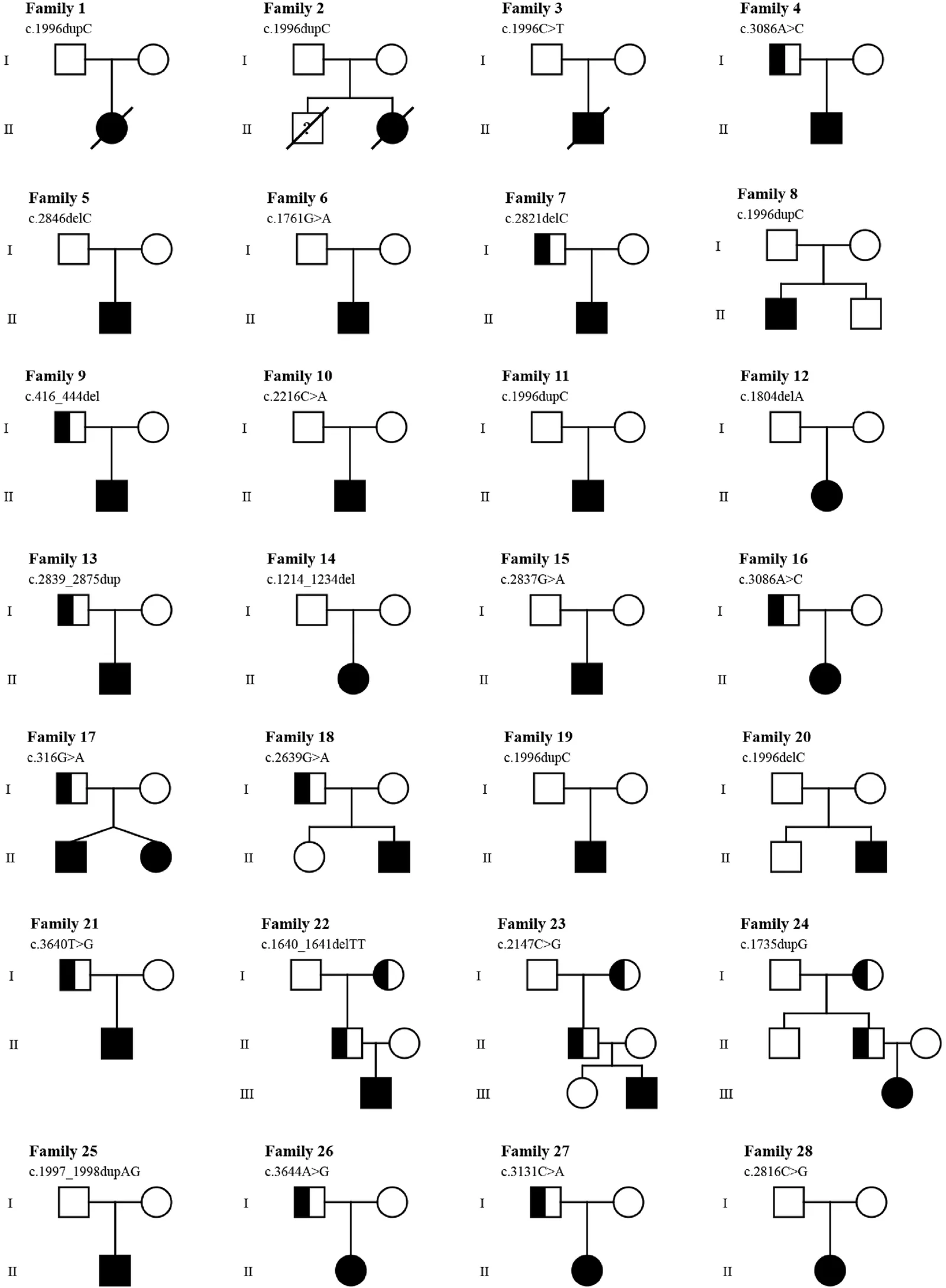
Pedigrees of the 28 families with MAGEL2-related disorders in our cohort. Squares represent males; circles represent females; black solid symbols indicate affected patients; empty symbols indicate unaffected subjects; the half black solid circles or squares indicate the variation carriers; a slash through the symbol means deceased; the square with a question mark denotes an untested male individual.

### 3.2 General clinical phenotypes

Table 1 summarizes the clinical symptoms of our patients. DD/ID, the typical symptoms of SYS, were universal in the cohort (28/28, 100%). Speech, motor, and cognitive delay were therefore the most consistent postnatal features. ASD or autistic features were documented in 6 of 10 evaluable individuals (60%). Seizures were reported in 7 of 22 evaluable individuals (32%), a frequency similar to which reported in the reference cohort by Mccarthy et al. (2018) (21/64, 33%).

**TABLE 1:** Clinical phenotypes of patients in our cohort.

Symptoms	Our cases	McCarthy et al. (5)
DD/ID	25/25 (100%)	70/70 (100%)
ASD diagnosis	6/10 (60%)	25/32 (78%)
Seizures	7/22 (32%)	21/64 (33%)
Sleep apnea	5/12 (42%)	38/50 (76%)
Respiratory distress	14/26 (54%)	41/58 (71%)
Feeding difficulties	22/25 (88%)	69/71 (97%)
Poor suck in infancy	22/24 (92%)	62/64 (97%)
Dysphagia	17/26 (65%)	48/57 (84%)
GERD	11/20 (55%)	34/60 (57%)
Hyperphagia	1/7 (14%)	14/56 (25%)
Chronic constipation	10/19 (53%)	39/55 (71%)
Neonatal hypotonia	23/26 (88%)	66/68 (97%)
Temperature instability	8/17 (47%)	40/60 (67%)
Contractures	14/25 (56%)	69/78 (88%)
Small hands	8/11 (73%)	12/14 (72%)
Small feet	5/8 (63%)	8/15 (61%)
Scoliosis	5/13 (38%)	26/46 (57%)
Short stature	7/11 (64%)	12/20 (60%)
GHD	5/7 (71%)	NA
Hypogonadism	11/19 (58%)	26/64 (41%)

Denominators vary because only evaluable individuals were included. Comparisons with McCarthy et al. are descriptive because of differences in age distribution, follow-up, and ascertainment methods.

Abbreviations: DD/ID, Developmental Delay/Intellectual Disability; ASD, autistic spectrum disorder; GERD, gastroesophageal reflux disease; GHD, growth hormone deficiency.

Neonatal and infantile feeding problems were prominent in this cohort. Feeding difficulties were present in 22 of 25 evaluable individuals (88%), poor suck in infancy in 22 of 24 (92%), dysphagia in 17 of 26 (65%), and gastroesophageal reflux disease (GERD) in 11 of 20 (55%). Hyperphagia was uncommon in the available records, being reported in 1 of 7 evaluable individuals (14%). Additionally, chronic constipation was documented in 53% of these individuals.

Respiratory involvement was a serious and early clinical manifestation. Respiratory distress was recorded in 54% of our patients. Sleep apnea was documented in 5 of 12 individuals (42%). In the majority of neonates and young infants, respiratory disease was accompanied by feeding failure, poor suck, dysphagia, or the need for tube feeding, supporting a clinically recognizable respiratory-feeding phenotype in early-onset SYS.

Muscle tone abnormalities and distal skeletal involvement were also frequent and characteristic. Neonatal hypotonia was recorded in 23 of 26 evaluable individuals (88%). Distal contractures were present in 14 of 25 individuals (56%), lower than the frequency reported by Mccarthy et al. (2018). Small hands, small feet, and scoliosis were observed in 73%, 63% and 38% of evaluable individuals, respectively.

Endocrine, growth, and genital abnormal findings were common among individuals with available data. Short stature was documented in 64% of our patients. Growth hormone (GH) levels were evaluated in 7 individuals, 5 of whom (71%) showed hyposecretion. Among them, four patients received recombinant human GH (rhGH) therapy, with significant improvements observed in height, muscle strength and endurance, as well as cognition and sociability. Besides, hypogonadism or genital abnormalities were present in 11 of 19 patients (58%). Temperature instability was noted in 8 of 17 individuals (47%).

These findings collectively indicate that the cohort recapitulates the core multisystem phenotype of SYS, while also emphasizing clinically important neonatal respiratory and feeding complications. The observed frequencies of most clinical features in our cohort were modestly lower than those reported by McCarthy et al. This difference may partly reflect the higher proportion of individuals carrying *MAGEL2* missense variants in our cohort, although differences in age distribution, ascertainment methods, follow-up duration, and evaluable denominators may also have contributed.

### 3.3 Genetic findings

Across the 28 individuals, 23 distinct *MAGEL2* gene variants were identified (Table 2). Figure 2 shows representative Sanger sequencing plots from 8 families. Fifteen individuals carried *de novo* variants, and 13 carried paternally inherited variants, consistent with the paternal expression and maternal imprinting of *MAGEL2* gene. These variants were predominantly clustered in the C-terminal region of MAGEL2 protein, expect for the p. Gly106Arg and p. Met139Asnfs*382 variants (Figure 3). The recurrent variant c.1996dupC, which has previously been reported to be a hot spot, was identified in 5 individuals. Additional variants affecting the same mutationally sensitive region included c.1996delC and c.1996C>T, which accounted for 2 individuals. Truncating variants in *MAGEL2* were confirmed in 19 patients. Missense mutations were identified in 8 patients, and in-frame deletion (p.Val405_Gln411del) were identified in 1 patient. Among the *MAGEL2* variants identified in our patients, 8 types (c.1996dupC, c.1996delC, c.1996C>T, c.1640_1641delTT, c.1761G>A, c.2639G>A, c.3086A>C, c.3131C>A) had been previously reported in the literature or recorded in public databases such as ClinVar, whereas the remaining 15 were not found in the available literature or database records, and were therefore considered novel variants.

**TABLE 2:** *MAGEL2* variants detected in this survey (NM_019066.5).

Nucleotide	Amino acid	Inheritance	N
c.316G>A	p.Gly106Arg	Paternally inherited	1
c.416_444del	p.Met139Asnfs*382	Paternally inherited	1
c.1214_1234del	p.Val405_Gln411del	*de novo*	1
c.1640_1641delTT	p.Pro547Argfs*165	Paternally inherited	1
c.1761G>A	p.Trp587*	*de novo*	1
c.1735dup	p.Ala579Glyfs*134	Paternally inherited	1
c.1804delA	p.Thr602Argfs*100	*de novo*	1
c.1996dupC	p.Gln666Profs*47	*de novo*	5
c.1996delC	p.Gln666Serfs*36	*de novo*	1
c.1996C>T	p.Gln666*	*de novo*	1
c.1997dupA	p.Gln667Alafs*46	*de novo*	1
c.2147C>G	p.Thr716Ser	Paternally inherited	1
c.2216C>A	p.Ser739*	*de novo*	1
c.2639G>A	p.Arg880His	Paternally inherited	1
c.2816C>G	p.Thr939Ser	*de novo*	1
c.2821delC	p.Arg941Valfs*3	Paternally inherited	1
c.2837G>A	p.Trp946*	*de novo*	1
c.2839_2875dup	p.Glu959Glyfs*4	Paternally inherited	1
c.2846delC	p.Pro949Leufs*7	*de novo*	1
c.3086A>C	p.Gln1029Pro	Paternally inherited	2
c.3131C>A	p.Ser1044*	Paternally inherited	1
c.3640T>G	p.Phe1214Val	Paternally inherited	1
c.3644A>G	p.His1215Arg	Paternally inherited	1

**FIGURE 2:**
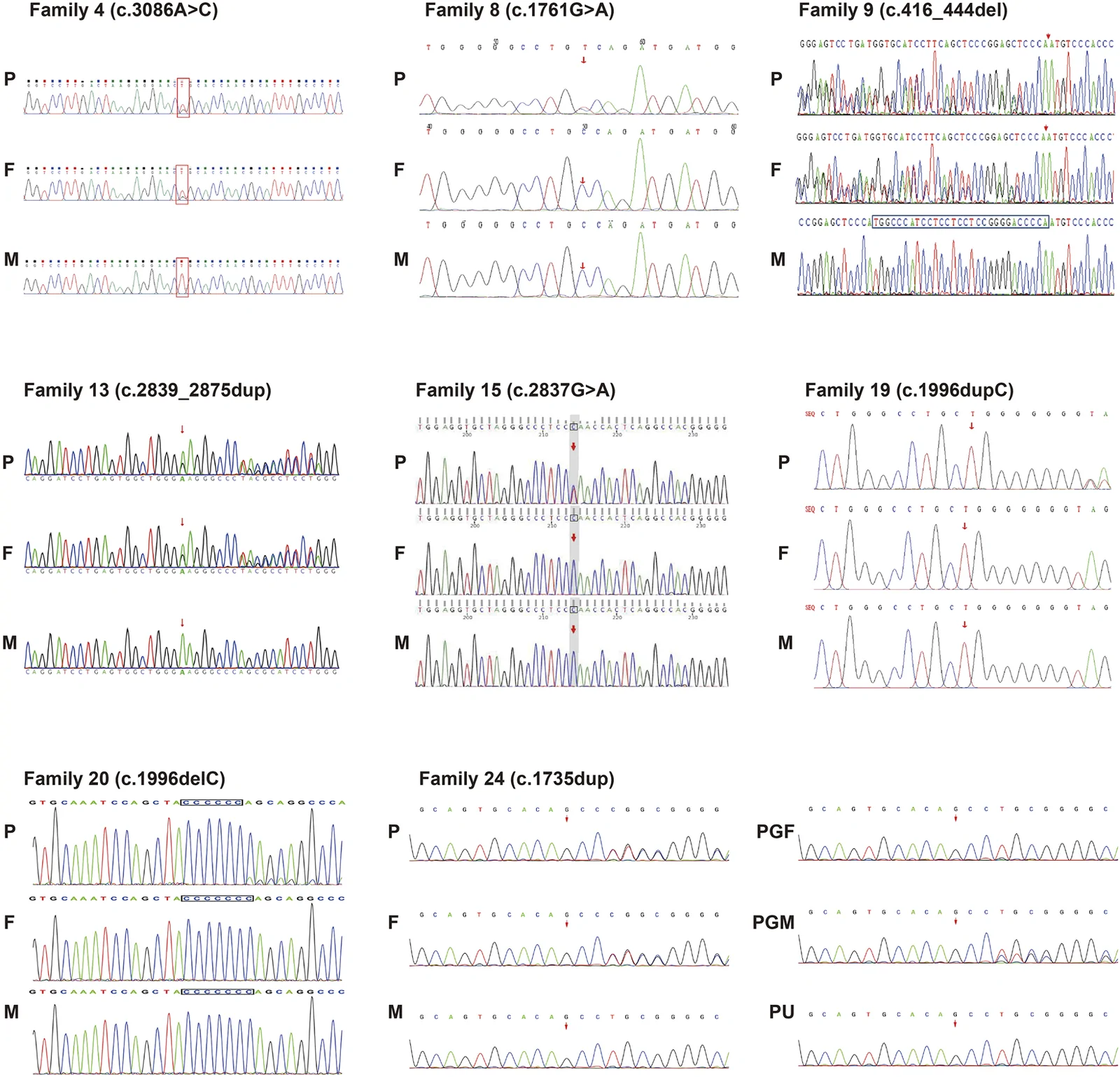
Electropherograms of Sanger sequencing of *MAGEL2* variants in affected individuals and family members. The c.3086A>C variant in Family 4, c.416_444del variant in Family 9, and c.2839_2875dup variant in Family 13 were heterozygous in the probands and their fathers, but absent from their mothers. The c.1761G>A variant in Family 8, c.2837G>A variant in Family 15, c.1996dupC variant in Family 19, and c.1996delC variant in Family 20 were detected only in the probands and were absent from both parents. In Family 24, the c.1735dup variant was present in the proband, her father, and her paternal grandmother, but absent from her mother, paternal grandfather, and paternal uncle, consistent with transmission from an unaffected father who inherited the variant from his mother, in keeping with the parent-of-origin effect of *MAGEL2*. Abbreviations: P, patient; F, father; M, mother; PGF, paternal grandfather; PGM, paternal grandmother; PU, paternal uncle.

**FIGURE 3:**
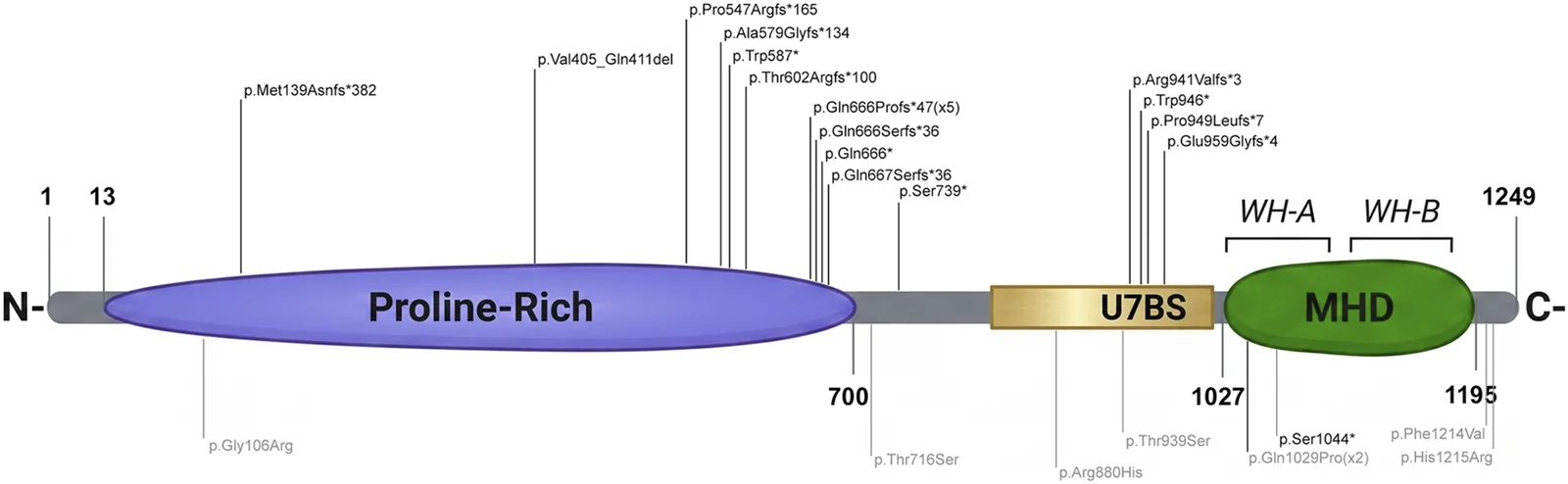
Schematic representation of the *MAGEL2* protein and the representative locations of disease-associated variants presented in this study. MHD, MAGE Homology Domain. The number in brackets indicates the number of individuals carrying recurrent variants. Atypical missense mutations are represented by gray color. All variants are described using the *MAGEL2* reference transcript NM_019066.5.

### 3.4 Critically ill and fatal cases

Four fatal cases were summarized separately (Table 3). Death occurred very early, at 5 days, 20 days, 32 days, and 98 days of life. Two affected individuals were female and another two were male. All the patients had apnea, neonatal hypotonia, respiratory distress, feeding difficulty, poor suck, dysphagia, distal contractures, dysmorphic features, and required intubation and mechanical ventilation. All required nasogastric or gastrostomy tube feeding. The two male infants presented with cryptorchidism. All the fatal cases carried *de novo* variants affecting c.1996: three had c.1996dupC and one had c.1996C>T. The leading cause of death in all four was respiratory distress.

**TABLE 3:** Clinical and genomic characteristics in fatal cases of Schaaf-Yang syndrome.

Characteristics	Case 1 (family 1)	Case 2 (family 2)	Case 3 (family 3)	Case 4 (family 19)
Age	20 d	98 d	5 d	32 d
Sex	F	F	M	M
IUGR	+	-	-	+
Fetal hypokinesia	+	-	-	+
Apnea	+	+	+	+
Neonatal hypotonia	+	+	+	+
Respiratory distress	+	+	+	+
Intubation	+	+	+	+
Mechanical ventilator	+	+	+	+
Feeding difficulties	+	+	+	+
Poor suck	+	+	+	+
Dysphagia	+	+	+	+
GERD	-	+	-	+
Use of NG/G tube	+	+	+	+
Contractures	+	+	+	+
Clubfoot	-	+	+	+
Dysmorphic features	+	+	+	+
Seizures	+	-	-	-
Cryptorchidism	NA	NA	+	+
CHD	+	+	+	-
Variation	c.1996dupC	c.1996dupC	c.1996C>T	c.1996dupC
Inheritance	*de novo*	*de novo*	*de novo*	*de novo*
Cause of death	Respiratory distress	Respiratory distress	Respiratory distress	Respiratory distress

Abbreviations: d, days; F, female; M, male; IUGR, intrauterine growth restriction; GERD, gastroesophageal reflux disease; CHD, congenital heart defects; +, present; -, not present; NA, not applicable.

One additional patient in our cohort also showed severe early-onset manifestations. The patient (Figure 1, Family 27) was a 5-month-old female infant carrying a paternally inherited *MAGEL2* c.3131C>A (p.Ser1044*) variant. Her mother had previously undergone termination of pregnancy for a 6-month fetus with cleft lip and palate. After birth, the patient had weak respiratory effort requiring ventilatory support, together with feeding difficulty needing nasogastric tube. She also exhibited finger joint contractures and congenital talipes equinovarus, and was later admitted to ICU because of aspiration pneumonia following choking during feeding. The outcomes of this infant remain unknown.

These cases delineate a severe neonatal SYS subgroup characterized by the convergence of respiratory failure, feeding impairment, hypotonia, and congenital contractures, particularly in association with *de novo* c.1996-region truncating variants.

### 3.5 Genotype-phenotype observations

Variants involving the c.1996 hotspot region appeared to be associated with a severe early-onset phenotype in several individuals. Among the 7 individuals carrying variants at c.1996 site, recurrent clinical features included neonatal or early-infantile presentation, feeding difficulty, respiratory distress, hypotonia, and distal contractures. The recurrent c.1996dupC variant was identified in 5 individuals and was repeatedly observed in patients with severe neonatal disease. Notably, three patients carrying c.1996dupC and one patient carrying c.1996C>T died due to respiratory failure during infancy.

Paternally inherited variants were identified in 13 individuals, underscoring the importance of parental testing and parent-of-origin interpretation for MAGEL2. In three cases, genetic testing was performed on the patients’ fathers and paternal grandmothers (Figure 1, Family 22–24), confirming that the variants carried by the phenotypically unaffected fathers were maternally inherited, consistent with the imprinting pattern of *MAGEL2*. Accordingly, the molecular diagnosis in SYS required not only variant detection but also assessment of inheritance and, when available, parental origin.

## 4 Discussion

To our knowledge, this is the first questionnaire-based survey focused on Chinese patients with SYS, aiming to delineate the clinical and molecular spectrum of this rare imprinting disorder in China. In this cohort of 28 individuals with MAGEL2-related disorders, the major clinical manifestations were consistent with the core phenotype reported in previous series (Schaaf et al., 2013; Fountain et al., 2017; Mccarthy et al., 2018), including DD/ID, neonatal hypotonia, feeding difficulties, respiratory distress, distal contractures and dysmorphic features. Thus, Chinese patients share the canonical multisystem SYS phenotype, while our data further emphasize early respiratory and feeding morbidity.

In our cohort, the most clinically salient finding was the high frequency of neonatal or early-infantile respiratory-feeding dysfunction. While SYS is classically defined as a Prader-Willi-like neurodevelopmental disorder caused by truncating variants of the paternally expressed *MAGEL2* gene (Schaaf et al., 2013; Tacer et al., 2017; Hoyos Sanchez et al., 2023), its initial presentation can be both nonspecific and severe. Patients with PWS caused by loss of the paternal 15q11-q13 region, including *MAGEL2*, often exhibit milder phenotypes than patients with SYS caused by truncating *MAGEL2* variants (Fountain et al., 2017; Dötsch et al., 2026), suggesting that SYS is not simply a loss-of-function disorder. One proposed explanation is that deletion of the entire paternal *MAGEL2* locus, including its regulatory elements, could allow leaky expression from the silent maternal allele, thereby partially compensating for the loss of the paternal copy (Matarazzo and Muscatelli, 2013). However, this mechanism cannot fully account for SYS pathogenesis. Since *MAGEL2* is a single-exon gene, transcripts harboring premature termination codons are predicted to evade nonsense-mediated decay, leading to the production of truncated MAGEL2 proteins (Hug et al., 2016; Lejeune, 2022). Truncating variants associated with SYS may exert effects beyond simple loss of function, possibly through toxic gain-of-function or neomorphic activities (Maaß et al., 2023; Fountain and Schaaf, 2016). For neonates presenting with unexplained respiratory compromise, poor feeding, hypotonia, and distal contractures, early *MAGEL2* testing should be considered, especially when Prader-Willi methylation testing is negative.

Our fatal cases further support the existence of a severe neonatal SYS subgroup. Four individuals died at early stage of life. All of them had neonatal hypotonia, feeding difficulties, distal contractures, respiratory distress, and required intubation and mechanical ventilation. The recorded cause of death was respiratory distress in all 4 infants. These findings in our patients are consistent with those reported in the Chinese SYS individuals. Huang et al., for instance, profiled 12 critically ill Chinese infants with SYS and identified respiratory failure as the leading cause of death during the neonatal period (Huang et al., 2023). In addition, Xiao et al. (2020) reported 5 children with SYS, all of whom died shortly after birth due to apnea or respiratory failure. In practice, for an infant with this constellation of findings, especially respiratory distress, we would recommend proactive respiratory monitoring, formal swallowing and nutritional assessments, and early, anticipatory guidance for the family.

Previously work identified the *MAGEL2* c.1990–c.1996 site as a recurrent hotspot, with c.1996dupC confirmed as the most common pathogenic variant (Fountain et al., 2017; Mccarthy et al., 2018). Consistently, five patients in our cohort carrying c.1996dupC tended to exhibit a more severe phenotype. The c.1996delC variant appears to be associated with the more severe end of the SYS phenotypic spectrum, including fetal hypokinesia, severe arthrogryposis, and perinatal lethality in some reported cases (Mejlachowicz et al., 2015). Paradoxically, a male patient in our cohort carrying a *de novo* c.1996delC variant showed relatively mild phenotypes, characterized by DD/ID, neonatal hypotonia, cryptorchidism, and chronic constipation, but without respiratory distress, joint contractures, or other severe manifestations. Reanalysis of the family sequencing data showed that this variant was present in a mosaic state, with 15/90 variant reads on WES and a low mutant peak on Sanger sequencing. This mosaicism may partly explain the relatively attenuated phenotype, because only a subset of cells is expected to carry the truncating *MAGEL2* variant. Given the imprinting pattern of MAGEL2, the clinical severity of a mosaic variant may depend on both the proportion and tissue distribution of cells carrying the affected paternal allele (Schubert and Schaaf, 2024; Cao et al., 2019). Therefore, a lower mosaic burden in disease-relevant tissues may reduce the amount of abnormal truncated MAGEL2 protein and partly explain the milder phenotype of this individual.

It is worth noting that 8 individuals in our cohort carried missense variants in *MAGEL2*. Compared with patients harboring truncating variants, these children showed overall milder phenotypes, and only one individual in our cohort showed distal contractures. In line with the observation from Patak et al. (2019), individuals carrying missense variants showed less overlap in both symptom burden and symptom profile. Nevertheless, these patients retained core SYS features, including universal DD/ID, dysmorphic facial features, mild feeding difficulties, and some also showed severe ASD traits. In light of the parent-of-origin effect of MAGEL2, segregation analysis further supported the clinical relevance of these variants, with 7 confirmed as paternally inherited, although one identified as *de novo*. We suspected that selected missense variants can perturb MAGEL2 function without producing the severe truncated-protein effect proposed for classic SYS (Pastucha et al., 2026; Castilla-Vallmanya et al., 2023). In line with current diagnostic recommendations, missense variants warrant cautious interpretation. They should not be considered equivalent to established pathogenic truncating variants without comprehensive analysis of segregation, rarity, phenotypic concordance, paternal-allele confirmation, and functional validation (Tacer et al., 2017; Patak et al., 2019). Taken together, these 8 individuals carrying *MAGEL2* missense variants were considered to have milder or atypical SYS. Future studies should assess the expression, stability, and subcellular localization of missense MAGEL2 proteins in patient-derived cells or relevant model systems. Functional evaluation of retromer/WASH-dependent endosomal recycling, cargo trafficking, protein interactions, and neuronal phenotypes will also be important to determine whether these variants alter MAGEL2 function and contribute to disease.

## 5 Conclusion

Several limitations should be acknowledged. The questionnaire-based and retrospective design inevitably limited direct, in-person phenotypic assessment and resulted in incomplete capture of some subtle clinical features. Moreover, although this study describes, to our knowledge, the first relatively large series of potentially pathogenic *MAGEL2* missense variants, the absence of functional validation limits definitive interpretation of their pathogenicity. The lack of standardized long-term follow-up is another limitation, as it prevented us from assessing longitudinal neurodevelopmental progression, endocrine trajectories, respiratory and feed-related outcomes, and age-dependent survival. Prospective natural-history studies with standardized follow-up protocols are therefore needed.

Nevertheless, the first nationwide questionnaire-based survey of SYS in China systematically characterized the clinical and molecular spectrum of Chinese MAGEL2-related disorders. Severe early-onset manifestations were observed in individuals carrying truncating variants affecting the c.1996 site, but this exploratory finding requires confirmation in larger, independently ascertained longitudinal cohorts. Our work highlights the need for earlier recognition of critically affected SYS patients, particularly those who may benefit from timely molecular diagnosis, intensified respiratory and nutritional surveillance, and coordinated multidisciplinary management in the early stage of life.

## Data Availability

The original raw sequencing data analyzed in this study were generated and retained by independent third-party diagnostic laboratories as part of routine clinical diagnostic testing and are not under the custody or control of the authors. Therefore, these raw sequencing files cannot be deposited by the authors in a public repository. The de-identified variant-level data supporting the findings of this study are included in the article. Further inquiries regarding the available data may be directed to the corresponding authors. Access to the original sequencing data, where available, would be subject to authorization by the respective diagnostic institutions and applicable ethical, privacy, and data-protection requirements.
